# The joint effects of inflammation and renal function status on in-hospital outcomes in patients with acute ischemic stroke treated with intravenous thrombolysis

**DOI:** 10.1186/s12883-024-04002-6

**Published:** 2024-12-31

**Authors:** Zhichao Huang, Xiaoyue Zhu, Xiuman Xu, Yi Wang, Yafang Zhu, Dongqin Chen, Yongjun Cao, Xia Zhang

**Affiliations:** 1 Department of Neurology and Clinical Research Center of Neurological Disease, the Second Affiliated Hospital of Soochow University, Sanxiang Road, Suzhou, Jiangsu Province 1055 China; 2 Department of Clinical Nutrition, Suzhou Municipal Hospital, Suzhou, China

**Keywords:** Acute ischemic stroke, Estimating glomerular filtration rate, White blood cell, C-reactive protein, Intravenous thrombolysis, Prognosis, Joint prediction

## Abstract

**Objective:**

We aimed to determine the predictive value of renal function status [estimating glomerular filtration rate (eGFR)] in conjunction with inflammatory biomarkers [white blood cell(WBC) and C-reactive protein (CRP)] for in-hospital outcomes in acute ischemic stroke (AIS) patients treated with intravenous thrombolysis (IVT).

**Methods:**

We retrospectively screened a total of 409 AIS patients treated with IVT. The study participants were classified into two groups according to post-stroke pneumonia or functional outcome. They were divided into four groups according to the cut-offs of inflammatory biomarkers and eGFR by receiver operating characteristics(ROC) curves for two outcomes of post-stroke pneumonia and functional status: WBC↓/eGFR↑, WBC↓/eGFR↓, WBC↑/eGFR↑, and WBC↑/eGFR↓for post-stroke pneumonia; and CRP↓/eGFR↑, CRP↓/eGFR↓, CRP↑/eGFR↑, and CRP↑/eGFR↓for functional outcome. Logistic regression models were used to calculate the odds ratios (ORs) and 95% confidence intervals (CIs) of post-stroke pneumonia or at-discharge functional outcome, using the WBC↓/eGFR↑group or CRP↓/eGFR↑group as the reference. The Net Reclassification Index (NRI) and the Integrated Discrimination Improvement (IDI) were calculated to analyze the combined predictive value.

**Results:**

Compared with patients in WBC↓/eGFR↑group, those in WBC↑/eGFR↑group had increased risk of post-stroke pneumonia (OR 5.15, 95% CI 1.67–15.87) and poor functional outcome (OR 5.95, 95% CI 2.25–15.74). Furthermore, patients in WBC↑/ eGFR↓group had the highest risk of clinical outcomes (all *P* value for trend < 0.001), the multivariable-adjusted ORs (95% CIs) were 7.04 (2.42–20.46) for post-stroke pneumonia and 8.64 (3.30–22.65) for poor functional outcome. The addition of WBC and eGFR to the basic model significantly improved risk prediction for post-stroke pneumonia (category-free NRI 69.0%, 95% CI 47.3%–90.7%; IDI 5.4%, 95% CI 2.6%–8.3%) and functional outcome (category-free NRI 59.4%, 95% CI 39.2%–79.9%; IDI 5.3%, 95% CI 2.9%–7.8%). Similarly, when we added CRP and eGFR to the basic model with conventional risk factors, the risk discrimination and prediction for post-stroke pneumonia and functional outcome was also significantly improved.

**Conclusion:**

Combining renal function status and inflammatory biomarkers within 4.5 h after onset could better predict in-hospital outcomes of AIS patients with IVT.

## Introduction

Intravenous thrombolytic therapy (IVT) with recombinant tissue plasminogen activator (rt-PA) has been shown to be the most effective treatment for acute ischemic stroke (AIS) patients within 4.5 h of the onset of symptoms [1], however, the prognosis for many patients receiving it is still poor due to the occurrence of complications. A number of studies have examined the association between poststroke medical complications and mortality. Pneumonia is the major early complication of stroke and is associated with high morbidity and mortality. Pneumonia by itself is also associated with longer hospital stays and poor functional outcomes [2, 3]. Meanwhile, more than one-third of stroke patients have renal dysfunction (CKD), presenting as a decrease of estimated glomerular filtration rate (eGFR, typically < 60 ml/min/1.73 m2) [4, 5]. Previous studies have shown that CKD is associated with poor prognosis, symptomatic intracranial hemorrhage (sICH) [3, 6] and death [7–10] in AIS patients. Both CKD and pneumonia are important factors contributing to the prognosis of AIS patients.

Moreover, inflammation plays an important role in ischemic stroke. Many inflammatory markers in peripheral blood are demonstrated to be correlated with ischemic stroke severity or prognosis. Furthermore, inflammation plays a critical role in the initiation and progression of CKD, and inflammation and CKD often co-exist in AIS patients. However, whether eGFR combined with inflammatory biomarkers can better predict the short-term prognosis of AIS patients has not been thoroughly investigated. This is important because a better understanding of risk factors and predictors of prognosis could enable clinicians to precisely identify patients at high risk and to give them appropriate treatments. Therefore in this paper, we aimed to determine the predictive value of eGFR and inflammatory biomarkers[white blood cell(WBC) or C-reactive protein (CRP)] for in-hospital outcomes in AIS patients treated with IVT.

### Patients and methods

This study retrospectively selected consecutive AIS patients who received intravenous rt-PA within 4.5 h of symptom onset from the Stroke registry at the Suzhou Comprehensive Stroke Center between May 2018 and May 2021. The inclusion criteria were based on indications for intravenous rt-PA thrombolysis [1]. The exclusion criteria were as follows: 1) patients with cerebral infarction sequelae and modified rankin scale (mRS) ≥ 1; 2) suffering from severe underlying diseases; 3) patients with stroke mimics; 4) missing mRS data; 5) patients without eGFR, WBC count, and CRP data collected within 4.5 h of symptoms of stroke onset. All patients were routinely given appropriate antithrombotic drugs and other conventional treatments 24 h after thrombolysis. The research program was approved by the Ethics Committee of the Second Affiliated Hospital of Soochow University and was conducted in accordance with the ethical standards set out in the 1964 Declaration of Helsinki and its later amendments (JDHG-2021–41). Informed consents were obtained from all participants or their caregivers.

Data on demographic characteristics, lifestyle, risk factors, medical history, clinical laboratory tests, and imagings were collected at admission. All information is obtained by trained staff through a standard management questionnaire. Baseline stroke severity was assessed by trained neurologists using the National Institutes of Health Stroke Scale (NIHSS) [11]. Oxfordshire Community Stroke Project (OCSP) classification [lacunar infarct (LACI), total anterior circulation infarcts (TACI), partial anterior circulation infarcts (PACI) and posterior circulation infarcts (POCI)], the Trial of Org 10,712 in Acute Stroke Treatment (TOAST) etiology [large artery atherosclerosis (LAA); cardioembolism (CE); small-artery occlusion (SAO); stroke of other determined cause (ODC)] were collected standardly. Blood samples were taken upon admission within 4.5 h of onset before IVT, and were tested using VITROS XT 3400 Chemistry Analyzer and Sysmex CS-5100 Coagulation Analyzer.

eGFR was calculated by the Chinese population Chronic Kidney Disease Epidemiology Collaboration (CKD-EPI) equation with an adjustment coefficient of 1.1:

The CKD-EPI estimate of renal function was calculated as recommended [12]: For women with a plasma creatinine ≤ 0.7, (plasma creatinine/0.7)−0.329 × (0.993)age (× 166 if black; × 144 if white or other); for women with a plasma creatinine > 0.7, (plasma creatinine/0.7)−1.209 × (0.993)age (× 166 if black; × 144 if white or other); for men with a plasma creatinine ≤ 0.9; (plasma creatinine/0.9) −0.411 × (0.993)age (× 163 if black; × 141 if white or other); for men with a plasma creatinine > 0.9, (plasma creatinine/0.9)−1.209 × (0.993)age (× 163 if black; × 141 if white or other).

Post-stroke pneumonia was diagnosed within the first 7 days after admission, with reference to Centers for Disease control and Prevention (CDC) criteria.

Post-stroke pneumonia was diagnosed within the first 7 days after admission, with reference to Centers for Disease control and Prevention (CDC) criteria [13]. Diagnosis of post-stroke pneumonia was according to the following criteria: new or aggravated cough and expectoration; an increase in respiratory rate of more than 22 times/min; fever (body temperature > 38 °C); decreased WBC count (< 4 × 109/L), increased WBC (> 11 × 109/L), or increased neutrophil ratio; audible moist rales; and abnormal chest radiology(patchy infiltration, lobar consolidation, or pleural effusion). If the patient presented three or more of the above conditions, we diagnosed the patient with post-stroke pneumonia.

The primary endpoint was at-discharge functional outcome, which was evaluated by the mRS at-discharge, and a mRS Score of 3–6 was defined as unfavorable functional outcome.

### Statistical analysis

Firstly, the study participants were classified into two groups in relation to post-stroke pneumonia status or functional outcome. The baseline characteristics of continuous variables were presented as means with SD or medians with interquartile ranges (IQRs), and were compared using Student’s t-test or Wilcoxon rank-sum test. The categorical variables were expressed as a percentage and were compared using a χ^2^ test.

In this study, we aimed to examine the combined effects of inflammatory biomarkers (WBC or CRP) and eGFR on the risk of post-stroke pneumonia and at-discharge functional outcome. Therefore, our participants were further divided into four groups in relation to inflammatory biomarkers and eGFR: WBC↓/eGFR↑, WBC↓/eGFR↓, WBC↑/eGFR↑, and WBC↑/eGFR↓; or CRP↓/eGFR↑, CRP↓/eGFR↓, CRP↑/eGFR↑, and CRP↑/eGFR↓. Logistic regression models were used to calculate the odds ratios (ORs) and 95% confidence intervals (CIs) of post-stroke pneumonia or at-discharge functional outcome, using the WBC↓/eGFR↑ group or CRP↓/eGFR↑ group as the reference. P trend < 0.05 indicated a significant change between groups. We constructed two logistic regression models: model 1 was an unadjusted logistic regression model; model 2 adjusted for age, sex, current smoking, admission NIHSS score, great vessels, medical history (hypertension, hyperglycemia, hyperlipidemia and coronary heart disease), history of stroke, ischemic stroke syndrome, use of anticoagulant and antiplatelet medication, duration of hospitalization.

We further evaluated the predictive ability of the joint effects of inflammation and renal function status by adding WBC and eGFR, or CRP and eGFR to the basic model with established risk factors. Basic model included age, sex, current smoking, admission NIHSS score, great vessels, medical history (hypertension, hyperglycemia, hyperlipidemia and coronary heart disease), history of stroke, ischemic stroke syndrome, use of anticoagulant and antiplatelet medication, duration of hospitalization. We calculated C statistics, the net reclassification index (NRI), and integrated discrimination improvement (IDI) to evaluate the risk discrimination and prediction for post-stroke pneumonia and functional outcome.

All *P* values were 2-tailed, and a significance level of 0.05 was used. Statistical analysis was conducted using SAS statistical software (version 9.4; SAS Institute, Cary, NC).

## Results

### Baseline clinical characteristics

**Fig. 1:**
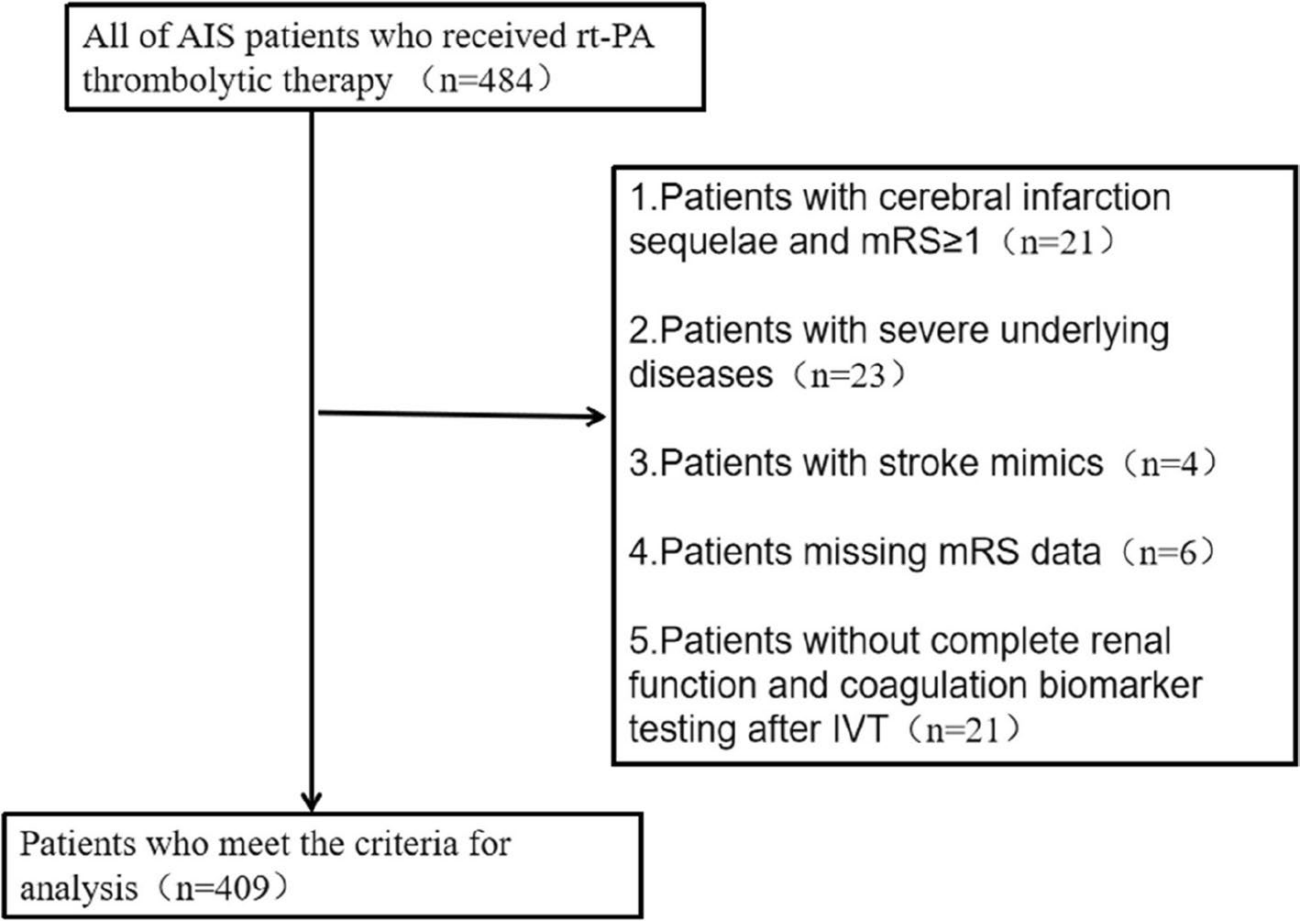
Patient flowchart

Figure 1 illustrated the flow chart of the patient inclusion process. After excluding patients with cerebral infarction sequelae and mRS ≥ 1(*n* = 21), those with severe underlying diseases(*n* = 23), stroke mimics (*n* = 4), and missing mRS data (*n* = 6), and those without complete renal function and coagulation biomarker testing after IVT (*n* = 21), a total of 409 patients were included in the final analysis. The baseline clinical characteristics of participants were shown in Table 1. Compared with patients in non-pneumonia group, those in pneumonia group were older and had higher admission NIHSS scores with higher proportion of large vessel occlusion and TACI, lower proportion of PACI and longer duration of hospitalization (all *P* < 0.05). The levels of WBC and CRP were higher, while that of eGFR were lower in pneumonia group(*P* < 0.05). The same results were observed in patients with poor functional outcomes compared to those with good ones(all *P* < 0.05).

**Table 1:** Baseline characteristics of study participants

	Post-stroke pneumonia			Functional outcome		
Characteristics^a^	Non-pneumonia	Pneumonia	***P*** value	Good	Poor	***P*** value
No. of patients	323 (79.0)	86 (21.0)		289 (70.7)	120 (29.3)	
Age, y	66.6 ± 12.8	73.1 ± 13.1	< 0.001	66.3 ± 12.9	71.9 ± 12.9	< 0.001
Male sex	189 (58.5)	44 (51.2)	0.22	176 (60.9)	57 (47.5)	0.01
Current cigarette smoking	116 (35.9)	25 (29.1)	0.24	109 (37.7)	32 (26.7)	0.03
Admission NIHSS score	3 (2–6)	12 (7–20)	< 0.001	3 (2–5)	11 (6–16)	< 0.001
Great vessels	45 (13.9)	51 (59.3)	< 0.001	32 (11.1)	64 (53.3)	< 0.001
Medical history						
Hypertension	208 (64.4)	59 (68.6)	0.47	180 (62.3)	87 (72.5)	0.05
Hyperglycemia	69 (21.4)	17 (19.8)	0.75	54 (18.7)	32 (26.7)	0.07
Hyperlipidemia	68 (21.1)	14 (16.3)	0.33	62 (21.5)	20 (16.7)	0.27
Coronary heart disease	21 (6.5)	6 (7.0)	0.87	18 (6.2)	9 (7.5)	0.64
History of stroke	44 (13.7)	13 (15.1)	0.73	42 (14.6)	15 (12.5)	0.58
Medications						
Anticoagulant	6 (1.9)	1 (1.2)	1.00	4 (1.4)	3 (2.5)	0.42
Antiplatelet	14 (4.3)	4 (4.7)	0.90	12 (4.2)	6 (5.0)	0.70
TOAST			< 0.001			< 0.001
LAA	252 (78.0)	46 (53.5)		226 (78.2)	72 (60.0)	
CE	18 (5.6)	3 (3.5)		19 (6.6)	2 (1.7)	
SAO	47 (14.6)	36 (41.9)		40 (13.8)	43 (35.8)	
ODS	6 (1.9)	1 (1.2)		4 (1.4)	3 (2.5)	
Stroke syndrome			< 0.001			< 0.001
TACI	37 (11.5)	39 (45.3)		27 (9.3)	49 (40.8)	
PACI	213 (65.9)	36 (41.9)		196 (67.8)	53 (44.2)	
POCI	63 (19.5)	11 (12.8)		56 (19.4)	18 (15.0)	
LACI	10 (3.1)	0		10 (3.5)	0	
Duration of hospitalization, d	8 (7–10)	13 (8–19)	< 0.001	8 (7–10)	11 (8–16)	< 0.001
WBC	7.1 ± 2.3	9.5 ± 3.4	< 0.001	6.9 ± 2.2	9.2 ± 3.3	< 0.001
CRP	6.9 ± 10.0	21.4 ± 31.4	< 0.001	7.4 ± 11.9	16.2 ± 26.4	< 0.001
eGFR	88.0 ± 20.3	80.5 ± 20.5	0.003	88.8 ± 17.6	80.7 ± 25.5	0.002

*Abbreviations NIHSS* National Institute of Health Stroke Scale, *LAA* Large-artery atherosclerosis, *CE* Cardioembolism, *SAO* Small-artery occlusion, *ODC* Stroke of other determined cause, *TACI* Total anterior circulation infarcts, *PACI* Partial anterior circulation infarcts, *POCI* Posterior circulation infarcts, *LACI* Lacunar infarcts, *WBC* White blood cell, *CRP* C-reactive protein, *eGFR* estimated glomerular filtration rate

^a^Continuous variables are expressed as mean ± standard deviation or median (interquartile range). Categorical variables are expressed as number (%)

### Joint effects of WBC and eGFR on in-hospital outcomes after IVT

**Fig. 2:**
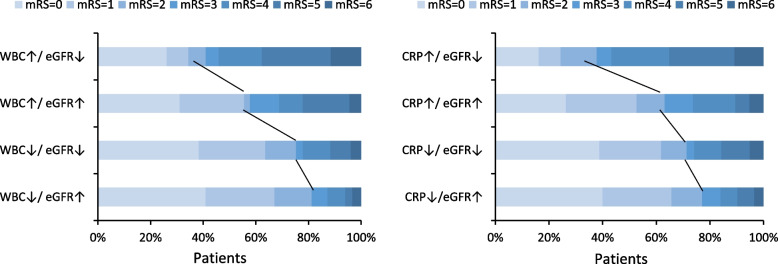
The joint effects of WBC,CRP and eGFR on clinical outcomes after ischemic stroke treated with IVT

According to the cutoffs of WBC and eGFR by ROC curves, the patients were divided into 4 groups. After adjustment for confounding factors including age, sex, NIHSS score, and medical history, compared with patients in WBC↓/eGFR↑ group, those in WBC↑/eGFR↑ group had increased risk of post-stroke pneumonia (OR 5.15, 95% CI 1.67–15.87) and poor functional outcome (OR 5.95, 95% CI 2.25–15.74). Furthermore, patients in WBC↑/ eGFR↓ group had the highest risk of clinical outcomes (all *P* value for trend < 0.001), the multivariable-adjusted ORs (95% CIs) were 7.04 (2.42–20.46) for post-stroke pneumonia and 8.64 (3.30–22.65) for poor functional outcome,the results were shown in Table 2 and Fig. 2.

**Table 2:** The joint effects of WBC and eGFR on clinical outcomes after ischemic stroke treated with IVT

	Groups by levels of baseline WBC and eGFR				
	WBC↓/ eGFR↑	WBC↓/ eGFR↓	WBC↑/ eGFR↑	WBC↑/ eGFR↓	*P* trend
Post-stroke Pneumonia					
No. (%) of cases	13 (8.7)	24 (15.5)	15 (34.1)	34 (56.7)	
Model 1	1.00	1.93 (0.93–3.95)	5.45 (2.34–12.68)	13.78 (6.42–29.60)	< 0.001
Model 2	1.00	0.90 (0.33–2.44)	5.15 (1.67–15.87)	7.04 (2.42–20.46)	< 0.001
Functional outcome					
No. (%) of cases	18 (12.1)	38 (24.7)	21 (46.7)	43 (70.5)	
Model 1	1.00	2.38 (1.29–4.40)	6.37 (2.96–13.69)	17.38 (8.31–36.38)	< 0.001
Model 2	1.00	1.69 (0.73–3.90)	5.95 (2.25–15.74)	8.64 (3.30–22.65)	< 0.001

Data are Odds ratios (95% confidence intervals)

Model 1: unadjusted logistic regression model

Model 2: adjusted for age, sex, current smoking, admission NIHSS score, great vessels, medical history (hypertension, hyperglycemia, hyperlipidemia and coronary heart disease), history of stroke, ischemic stroke syndrome, use of anticoagulant and antiplatelet medication, duration of hospitalization

*Abbreviations*: *WBC* White blood cell, *eGFR* estimated glomerular filtration rate

### Joint effects of CRP and eGFR on in-hospital outcomes after IVT

Table 3 and Fig. 2 shows the relationships between CRP and eGFR with post-stroke pneumonia and functional outcome in patients with acute ischemic stroke treated with IVT. Patients in CRP↑/ eGFR↓ group had the highest risk of clinical outcomes (all P value for trend < 0.001). The ORs (95% CIs) of CRP↑/ eGFR↓ group were 7.90 (4.04–15.46) for post-stroke pneumonia and 12.54 (5.51–28.56) for poor functional outcome, compared with those in CRP↓/eGFR↑ group. After adjustment for age, sex, NIHSS score, medical history, and other covariates in model 2, the ORs (95% CIs) were 4.76 (1.73–13.10) for post-stroke pneumonia and 5.51 (1.95–15.55) for poor functional outcome after acute ischemic stroke.

**Table 3:** The joint effects of CRP and eGFR on clinical outcomes after ischemic stroke treated with IVT

	Groups by levels of baseline CRP and eGFR				
	CRP↓/eGFR↑	CRP↓/ eGFR↓	CRP↑/ eGFR↑	CRP↑/ eGFR↓	*P* trend
Post-stroke Pneumonia					
No. (%) of cases	20 (12.8)	22 (14.9)	8 (21.1)	36 (53.7)	
Model 1	1.00	1.19 (0.62–2.28)	1.81 (0.73–4.51)	7.90 (4.04–15.46)	< 0.001
Model 2	1.00	0.57 (0.21–1.58)	1.18 (0.38–3.66)	4.76 (1.73–13.10)	< 0.001
Functional outcome					
No. (%) of cases	31 (17.7)	54 (30.3)	8 (42.1)	27 (73.0)	
Model 1	1.00	2.02 (1.22–3.34)	3.38 (1.26–9.09)	12.54 (5.51–28.56)	< 0.001
Model 2	1.00	1.73 (0.80–3.73)	1.75 (0.56–5.43)	5.51 (1.95–15.55)	0.002

Data are Odds ratios (95% confidence intervals)

Model 1: unadjusted logistic regression model

Model 2: adjusted for age, sex, current smoking, admission NIHSS score, great vessels, medical history (hypertension, hyperglycemia, hyperlipidemia and coronary heart disease), history of stroke, ischemic stroke syndrome, use of anticoagulant and antiplatelet medication, duration of hospitalization

*Abbreviations CRP* C-reactive protein, *eGFR* estimated glomerular filtration rate

### Predictive ability of inflammatory biomarkers and eGFR on study outcomes after ischemic stroke treated with IVT

**Fig. 3:**
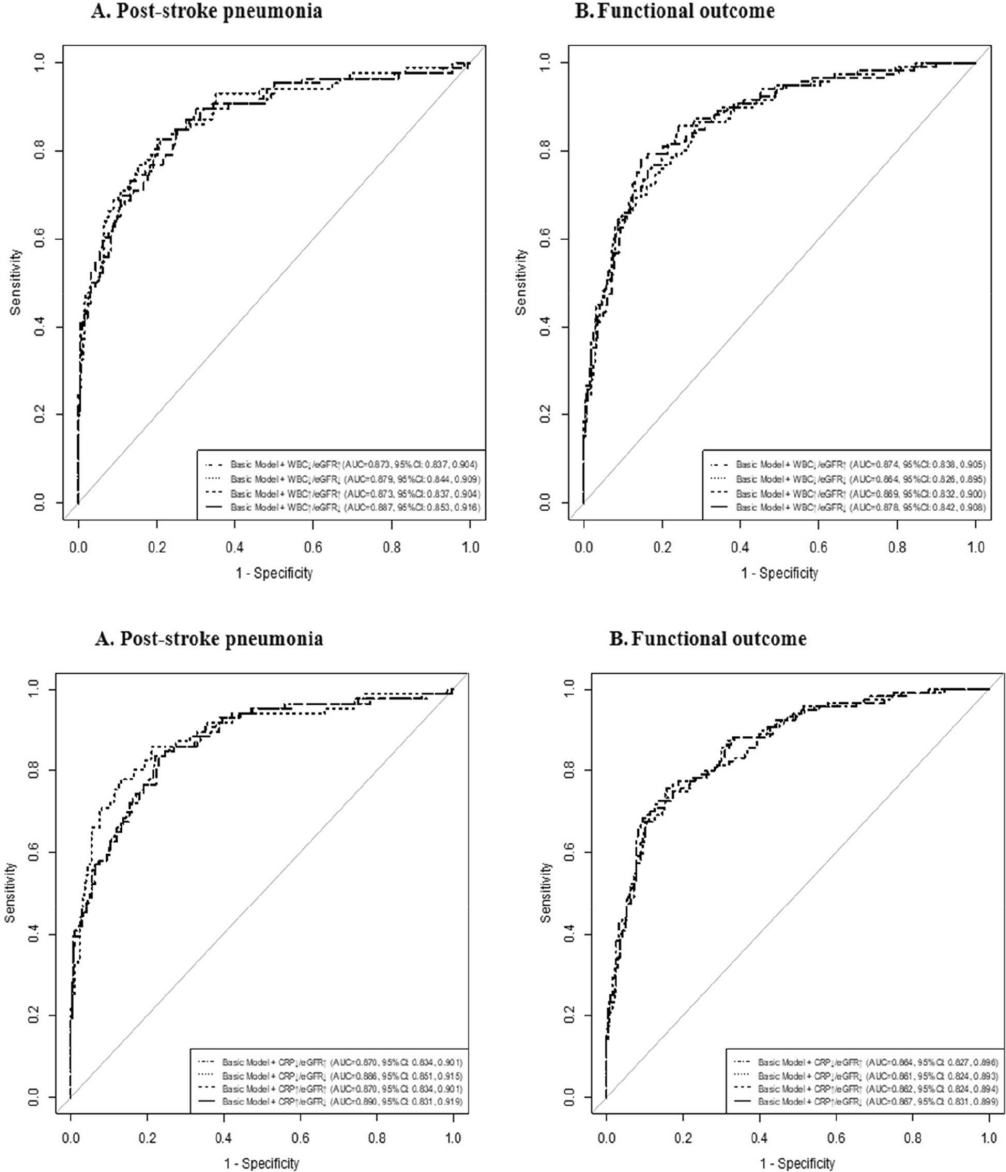
Performance of models with inflammatory biomarkers and eGFR for clinical outcomes after ischemic stroke treated with IVT

We further assessed the incremental prediction utility of the combination of inflammatory biomarkers and eGFR beyond the basic model for clinical outcomes. The basic model included age, sex, NIHSS score, medical history, ischemic stroke syndrome and conventional risk factors. The fourth group demonstrated the highest predictive value, having the largest auc after adding on to the base model(Fig. 3), as C statistics increased from 0.870 (95% CI: 0.834–0.901) to 0.889 (95% CI: 0.855–0.918) for post-stroke pneumonia, and from 0.861 (95% CI: 0.824–0.893) to 0.887 (95% CI: 0.852–0.916) for functional outcome. Furthermore, as shown in Table 4, the addition of WBC and eGFR to the basic model significantly improved risk prediction for post-stroke pneumonia (category-free NRI 69.0%, 95% CI 47.3%–90.7%; IDI 5.4%, 95% CI 2.6%–8.3%) and functional outcome (category-free NRI 59.4%, 95% CI 39.2%–79.9%; IDI 5.3%, 95% CI 2.9%–7.8%). Similarly, when we added CRP and eGFR to the basic model with conventional risk factors, the risk discrimination and prediction for post-stroke pneumonia and functional outcome was also significantly improved.

**Table 4:** Performance of models with inflammatory biomarkers and eGFR for clinical outcomes after ischemic stroke treated with IVT

	NRI (category free)		IDI	
	Estimate (95% CI), %	*P* value	Estimate (95% CI), %	*P* value
Post-stroke Pneumonia				
Basic model	Reference		Reference	
Basic model + WBC + eGFR	69.0 (47.3–90.7)	< 0.001	5.4 (2.6–8.3)	< 0.001
Basic model + CRP + eGFR	71.3 (49.1–93.4)	< 0.001	8.4 (4.2–12.6)	< 0.001
Functional outcome				
Basic model	Reference		Reference	
Basic model + WBC + eGFR	59.4 (39.2–79.7)	< 0.001	5.3 (2.9–7.8)	< 0.001
Basic model + CRP + eGFR	27.5 (71.3–47.9)	0.01	2.8 (0.8–4.9)	0.008

Basic model included age, sex, current smoking, admission NIHSS score, great vessels, medical history (hypertension, hyperglycemia, hyperlipidemia and coronary heart disease), history of stroke, ischemic stroke syndrome, use of anticoagulant and antiplatelet medication, duration of hospitalization

*Abbreviations: WBC* White blood cell, *CRP* C-reactive protein, *eGFR* estimated glomerular filtration rate, *CI* Confidence interval, *IDI* Integrated discrimination improvement, *NRI* Net reclassification index

## Discussion

In this study, we demonstrated that inflammation biomarkers and eGFR are important factors affecting in-hospital outcomes of patients with AIS after IVT treatment, and patients with WBC↑/ eGFR↓ and CRP↑/ eGFR↓ had the worst post-stroke pneumonia and poor functional outcomes compared with other groups. In addition, the addition of inflammatory biomarkers (WBC or CRP) combined with eGFR to the basic model with conventional risk factors significantly improved risk prediction for post-stroke pneumonia and functional outcome.

Previous studies have shown that CKD and stroke have similar risk factors and pathophysiological mechanisms, and low eGFR is considered to be an independent risk factor for cardiovascular and cerebrovascular events and death in stroke patients [14, 15]. Cohort studies and meta-analyses have shown that eGFR reduction increases stroke risk by approximately 40% [16], and the presence of proteinuria increases stroke risk by up to 70% [17], even after adjusting for traditional cardiovascular risk factors. In this study, we determined the effect of renal function status on in-hospital clinical outcomes in AIS patients with rt-PA, similar to ENCHANTED's postmortem findings that baseline renal function status was associated with an increased 90-day risk of death [7]. While the exact mechanism by which eGFR reduction leads to harmful effects is not fully understood, it appears to be a multifactorial process. First, traditional cardiovascular risk factors, including aging, high blood pressure, smoking, and dyslipidemia, were more prevalent in patients with CKD. Secondly, the greater burden of cardiovascular and cerebrovascular diseases caused by CKD may lead to poor peripheral collateral circulation and increased infarct volume, thus hindering vascular recanalization after stroke and leading to worse prognosis [18]. Third, non-conventional risk factors directly caused by kidney disease, such as chronic inflammation, oxidative stress and thrombosis factors, are also considered to be the cause of excessive cerebrovascular risk in patients with CKD, which can lead to vascular damage and endothelial dysfunction [19, 20].

In addition, CKD often alters the nature and structure of fibrin clots, which may lead to greater resistance to rt-PA thrombolysis [21]. CKD may also counteract the effects of rt-PA by impacting endothelial cell release of tissue-type plasminogen activators, thereby reducing endothelial thrombolysis [22]. Therefore, for patients with cerebral infarction, it is feasible to actively improve renal function and thus be more beneficial to clinical prognosis. In addition, the effect of gender on the results cannot be ruled out. A recent study showed that the early prognosis of women with cardiac embolism is worse than that of men [23]. We also found similar results, with higher rates in men among patients with a good prognosis. However, we found an interesting result: Among patients with a good prognosis, the proportion of current smokers was higher. This may be due to the small sample size of our study. In addition, we observed a higher incidence of pneumonia and a poorer prognosis in patients with large vessel occlusion.

Our study also confirmed the predictive value of inflammatory factors (WBC and CRP) combined with eGFR in in-hospital prognosis of patients with acute cerebral infarction, and patients in WBC↑/ eGFR↓ and CRP↑/ eGFR↓ groups had the worst short-term functional prognosis. It was previously believed that highly sensitive C-reactive protein (hs-CRP) can effectively reflect the inflammatory status of the body, and has a high sensitivity to mild aseptic inflammation, and has been widely used in clinical detection of inflammation in various diseases [24]. In patients with CKD, inflammatory biomarkers, including interleukin-6 (IL-6), tumor necrosis factor alpha (TNF-α), CRP, fibrinogen, and serum albumin, have been shown to be independently associated with atherosclerotic events and death, suggesting that inflammation may play a key role in cardiovascular events and stroke in patients with CKD [25]. Inflammatory cell damage in patients with CKD can also be demonstrated by the correlation between thrombomodulin (TM) and eGFR. TM is a vascular protective transmembrane glycoprotein with anticoagulant and anti-inflammatory activities [26]. It can be released and shed from the endothelium as an extracellular soluble form, leading to inflammatory cell damage, while studies have shown a moderate association between TM and eGFR, even after adjusting for age. All the data implied a mutual role between inflammation and renal function status.

Previous studies have shown that hypertension patients with cerebral infarction ≥ 1.5cm in diameter complicated with cerebral infarction have higher levels of inflammation-related factors in the body after onset, and hs-CRP and IL-6 are positively correlated with cerebral infarction diameter, which indicated the larger the diameter of cerebral infarction, the more obvious the inflammatory response of the body. 2 h after a cerebral infarction, significant pathophysiological changes occur in the brain tissue, leading to elevation of inflammation in the body [27]. With the increase of infarct size, especially in the area of cerebral ischemia and hypoxia, the above pathophysiological process became more obvious [28, 29]. Moreover, a large number of CRPs produced will further activate the complement system of the body, thus aggravating the damage of vascular endothelial cells, activating the coagulation system [30], leading to further expansion of thrombus [31], aggravating the progression of the disease and the disturbance of local blood circulation, resulting in poor prognosis [32]. Our results were consistent with the above conclusions, and the baseline NIHSS score and the proportion of large blood vessel occlusion in patients with pneumonia were significantly higher than those without pneumonia.

Our study has the following limitations. First, the prevalence of CKD in this study may have been overestimated because eGFR was estimated based on baseline creatinine at the time of vascular events, and acute kidney injury is a common complication after stroke [10]. Second, the majority of patients included in the non-inflammatory group had mild stroke with a baseline NIHSS score of 3, which is generally not associated with many systemic sequelae. Third, only one measurement was performed for each parameter, which may weaken the relevance of the study results to some extent.

## Conclusions

In this study, we demonstrated that elevated WBC or CRP levels combined with low eGFR were associated with highest risk of post-stroke pneumonia and unfavorable at-discharge functional outcome in AIS patients after IVT. The coexistence of inflammation and renal dysfunction improved risk prediction for clinical outcomes after acute ischemic stroke. In future studies, it is necessary to study the change trend of inflammatory factors and renal function in AIS patients after admission and their relationship with functional prognosis.

## Data Availability

All data generated or analysed during this study are included in this article. Further enquiries can be directed to the corresponding author.

## References

[1] Jauch EC, Saver JL, Adams HP Jr, et al. Guidelines for the early management of patients with acute ischemic stroke: a guideline for healthcare professionals from the American Heart Association/American Stroke Association. Stroke. 2013;44:870–947. 23370205 10.1161/STR.0b013e318284056a

[2] Ingeman A, Andersen G, Hundborg HH, et al. In-hospital medical complications, length of stay, and mortality among stroke unit patients. Stroke. 2011;42(11):3214–8. 21868737 10.1161/STROKEAHA.110.610881

[3] Koennecke HC, Belz W, Berfelde D, et al. Factors influencing in-hospital mortality and morbidity in patients treated on a stroke unit. Neurology. 2011;77(10):965–72. 21865573 10.1212/WNL.0b013e31822dc795

[4] Di Angelantonio E, Chowdhury R, Sarwar N, et al. Chronic kidney disease and risk of major cardiovascular disease and non-vascular mortality: prospective population based cohort study. BMJ. 2010;341: c4986. 20884698 10.1136/bmj.c4986 PMC2948649

[5] Rowat A, Graham C, Dennis M. Renal dysfunction in stroke patients: a hospital-based cohort study and systematic review. Int J Stroke. 2014;9:633–9. 24621343 10.1111/ijs.12264

[6] Jung JM, Kim HJ, Ahn H, et al. Chronic kidney disease and intravenous thrombolysis in acute stroke: a systematic review and meta-analysis. J Neurol Sci. 2015;358:345–50. 26434615 10.1016/j.jns.2015.09.353

[7] Carr SJ, Wang X, Olavarria VV, ENCHANTED Investigators, et al. Influence of renal impairment on outcome for thrombolysis-treated acute ischemic stroke: ENCHANTED (Enhanced Control of Hypertension and Thrombolysis Stroke Study) post hoc analysis. Stroke. 2017;48:2605–9. 28739832 10.1161/STROKEAHA.117.017808

[8] Gadalean F, Simu M, Parv F, et al. The impact of acute kidney injury on inhospital mortality in acute ischemic stroke patients undergoing intravenous thrombolysis. PLoS ONE. 2017;12: e0185589. 29040276 10.1371/journal.pone.0185589 PMC5645137

[9] Laible M, Mohlenbruch MA, Pfaff J, et al. Influence of renal function on treatment results after stroke thrombectomy. Cerebrovasc Dis. 2017;44:351–8. 29084408 10.1159/000481147

[10] Zorrilla-Vaca A, Ziai W, Connolly ES Jr, et al. Acute kidney injury following acute ischemic stroke and intracerebral hemorrhage: a meta-analysis of prevalence rate and mortality risk. Cerebrovasc Dis. 2018;45:1–9. 29176313 10.1159/000479338

[11] Goyal M, Menon BK, van Zwam WH, et al. Endovascular thrombectomy after large-vessel ischaemic stroke: a meta-analysis of individual patient data from five randomised trials. Lancet. 2016;387:1723–31. 26898852 10.1016/S0140-6736(16)00163-X

[12] Levey AS, Stevens LA, Schmid CH, et al. A new equation to estimate glomerular filtration rate [published correction appears in Ann Intern Med. 2011 Sep 20;155(6):408]. Ann Intern Med 2009;150(9):604–612. 10.7326/0003-4819-150-9-200905050-00006 PMC2763564 19414839

[13] Horan TC, Andrus M, Dudeck MA. CDC/NHSN surveillance definition of health care-associated infection and criteria for specific types of infections in the acute care setting [published correction appears in Am J Infect Control. 2008 Nov;36(9):655]. Am J Infect Control 2008;36(5):309–332. 10.1016/j.ajic.2008.03.002 18538699

[14] Gensicke H, Frih AA, Strbian D, the Thrombolysis in Stroke Patients (TRISP) Collaborators, et al. Prognostic significance of proteinuria in stroke patients treated with intravenous thrombolysis. Eur J Neurol. 2017;24:262–9. 27862667 10.1111/ene.13179

[15] Lee M, Saver JL, Chang K-H, et al. Low glomerular filtration rate and risk of stroke: meta-analysis. BMJ. 2010;341: c4249. 20884696 10.1136/bmj.c4249 PMC2948650

[16] Ninomiya T, Perkovic V, Verdon C, et al. Proteinuria and stroke: a meta-analysis of cohort studies. Am J Kidney Dis. 2009;53:417–25. 19070947 10.1053/j.ajkd.2008.08.032

[17] Yang B, Zhu J, Miao Z, et al. Cystatin C is an independent risk factor and therapeutic target for acute ischemic stroke. Neurotox Res. 2015;28:1–7. 25697425 10.1007/s12640-015-9522-3

[18] Shuaib A, Butcher K, Mohammad AA, et al. Collateral blood vessels in acute ischaemic stroke: a potential therapeutic target. Lancet Neurol. 2011;10:909–21. 21939900 10.1016/S1474-4422(11)70195-8

[19] Sjøland JA, Sidelmann JJ, Brabrand M, et al. Fibrin clot structure in patients with end-stage renal disease. Thromb Haemost. 2007;98:339–45. 17721616

[20] Hrafnkelsdóttir T, Ottosson P, Gudnason T, et al. Impaired endothelial release of tissue-type plasminogen activator in patients with chronic kidney disease and hypertension. Hypertension. 2004;44:300–4. 15249548 10.1161/01.HYP.0000137380.91476.fb

[21] Toyoda K, Ninomiya T. Stroke and cerebrovascular diseases in patients with chronic kidney disease. Lancet Neurol. 2014;13:823–33. 25030514 10.1016/S1474-4422(14)70026-2

[22] Tonelli M, Karumanchi SA, Thadhani R. Epidemiology and mechanisms of uremia-related cardiovascular disease. Circulation. 2016;133:518–36. 26831434 10.1161/CIRCULATIONAHA.115.018713

[23] Inogés M, Arboix A, García-Eroles L, et al. Gender Predicts Differences in Acute Ischemic Cardioembolic Stroke Profile: Emphasis on Woman-Specific Clinical Data and Early Outcome—The Experience of Sagrat Cor Hospital of Barcelona Stroke Registry. Medicina. 2024;60:101. 38256361 10.3390/medicina60010101 PMC10819324

[24] Towfighi A, Cheng EM, Ayala-Rivera M, et al. Randomized controlled trial of a coordinated care intervention to improve risk factor control after stroke or transient ischemic attack in the safety net: Secondary stroke prevention by Uniting Community and Chronic care model teams Early to End Disparities (SUCCEED). BMC Neurol. 2017;17:24. 28166784 10.1186/s12883-017-0792-7 PMC5294765

[25] Amdur RL, Feldman HI, Dominic EA, et al. Use of measures of inflammation and kidney function for prediction of atherosclerotic vascular disease events and death in patients with ckd: findings from the cric study. Am J Kidney Dis. 2019;73:344–53. 30545708 10.1053/j.ajkd.2018.09.012 PMC6812505

[26] Martin FA, Murphy RP, Cummins PM. Thrombomodulin and the vascular endothelium: insights into functional, regulatory, and therapeutic aspects. Am J Physiol Heart Circ Physiol. 2013;304:H1585–97. 23604713 10.1152/ajpheart.00096.2013 PMC7212260

[27] Fluri F, Grünstein D, Cam E, et al. Fullerenols and glucosamine fullerenes reduce infarct volume and cerebral inflammation after ischemic stroke in normotensive and hypertensive rats. Exp Neurol. 2015;265:142–51. 25625851 10.1016/j.expneurol.2015.01.005

[28] Sato K, Yamashita T, Kurata T, et al. Telmisartan ameliorates inflammatory responses in SHR-SR after tMCAO. J Stroke Cerebrovasc Dis. 2014;23:2511–9. 25245484 10.1016/j.jstrokecerebrovasdis.2014.02.019

[29] Moller K, Boltze J, Pösel C, et al. Sterile inflammation after permanent distal MCA occlusion in hypertensive rats. J Cereb Blood Flow Metab. 2014;34:307–15. 24220169 10.1038/jcbfm.2013.199 PMC3915208

[30] Min LJ, Mogi M, Tsukuda K, et al. Direct stimulation of angiotensin II type 2 receptor initiated after stroke ameliorates ischemic brain damage. Am J Hypertens. 2014;27:1036–44. 24572705 10.1093/ajh/hpu015

[31] Gurbuzer N, Gozke E, Ayhan BZ. Gamma-glutamyl transferase levels in patients with acute ischemic stroke. Cardiovasc Psychiatry Neurol. 2014;20:170–6. 10.1155/2014/170626 PMC4151543 25202453

[32] Pires PW, Girgla SS, Moreno G, et al. Tumor necrosis factor-a inhibition attenuates middle cerebral artery remodeling but increases cerebral ischemic damage in hypertensive rats. Am J Physiol Heart Circ Physiol. 2014;307:H658–69. 25015967 10.1152/ajpheart.00018.2014 PMC4280151

